# Long term follow-up of patients with newly diagnosed glioblastoma treated by intraoperative photodynamic therapy: an update from the INDYGO trial (NCT03048240)

**DOI:** 10.1007/s11060-024-04693-4

**Published:** 2024-05-16

**Authors:** Iulia Peciu-Florianu, Quentin Vannod-Michel, Enora Vauleon, Marie-Edith Bonneterre, Nicolas Reyns

**Affiliations:** 1 Neurosurgery Department, CHU-Lille, F-59000 Lille, France; 2 U1189-ONCO-THAI-Assisted Laser Therapy and Immunotherapy for Oncology, University of Lille, INSERM, CHU-Lille, F-59000 Lille, France; 3 Neuroradiology Department, CHU-Lille, F-59000 Lille, France; 4 Neuro-Oncology Department, CHU-Lille, F-59000 Lille, France; 5 Hemerion Therapeutics, Villeneuve D’Ascq, France

**Keywords:** Glioblastoma, Intraoperative photodynamic therapy, Follow-up, Survival

## Abstract

**Purpose:**

Glioblastoma remains incurable despite optimal multimodal management. The interim analysis of open label, single arm INDYGO pilot trial showed actuarial 12-months progression-free survival (PFS) of 60% (median 17.1 months), actuarial 12-months overall survival (OS) of 80% (median 23.1 months). We report updated, exploratory analyses of OS, PFS, and health-related quality of life (HRQOL) for patients receiving intraoperative photodynamic therapy (PDT) with 5-aminolevulinic acid hydrochloride (5-ALA HCl).

**Methods:**

Ten patients were included (May 2017 – April 2021) for standardized therapeutic approach including 5-ALA HCl fluorescence-guided surgery (FGS), followed by intraoperative PDT with a single 200 J/cm^2^ dose of light. Postoperatively, patients received adjuvant therapy (Stupp protocol) then followed every 3 months (clinical and cerebral MRI) and until disease progression and/or death. Procedure safety and toxicity occurring during the first four weeks after PDT were assessed. Data concerning relapse, HRQOL and survival were prospectively collected and analyzed.

**Results:**

At the cut-off date (i.e., November 1st 2023), median follow-up was 23 months (9,7–71,4). No unacceptable or unexpected toxicities and no treatment-related deaths occurred during the study. Kaplan–Meier estimated 23.4 months median OS, actuarial 12-month PFS rate 60%, actuarial 12-month, 24-month, and 5-year OS rates 80%, 50% and 40%, respectively. Four patients were still alive (1 patient free of recurrence).

**Conclusion:**

At 5 years-follow-up, intraoperative PDT with surgical maximal excision as initial therapy and standard adjuvant treatment suggests an increase of time to recurrence and overall survival in a high proportion of patients. Quality of life was maintained without any severe side effects.

**Trial registration NCT number:**

NCT03048240. EudraCT number: 2016–002706-39.

## Introduction

### Background

Glioblastoma Multiforme (GBM) is an aggressive primary brain tumour with a particularly dismal prognosis. The incidence of glioblastoma multiforme ranges up to 5 per 100,000 people and is increasing in many countries [1]. The disease often progresses rapidly, with growth patterns that vary widely between patients translating its important heterogenicity [2]. Studies describe a growth rate of 1.4% per day and a volume doubling time of 49.6 days [3, 4]. GBM is an aggressive and infiltrative tumour, with microscopic extension into normal brain parenchyma. Microscopic, infiltrating cells can be found centimetres away from the edge of the visible tumour mass [5].

The standard of care (SOC) [5–7] includes maximal surgical resection whenever possible, followed by radiotherapy (RT), and chemotherapy (Temozolomide®, TMZ) e.g., the Stupp protocol. However, complete resection is rarely achievable because isolated tumour cells usually infiltrate the surrounding brain, leading to inevitable recurrence within 2 cm of the tumor bed in 85% of cases [4, 8] and a median progression free survival below 12 months. The median overall survival is thus under 16 months and the 2-and 5-year median survival rates are around 18.6% and 5.8%, respectively [9].

Photodynamic therapy (PDT) is based on the photochemical reaction induced by exposure of photosensitized cells to light, at a specific wavelength. For high-grade gliomas (HGG), 5 aminolevulinic acid hydrochloride (5-ALA HCl) can be used as a photosensitizer precursor that is metabolized to a photosensitizer e.g., protoporphyrin IX (PpIX). Exogenous exposure to 5-ALA HCl leads to a specific accumulation of PpIX in tumours cells with a peak 6–8 h after 5-ALA HCl ingestion [10]. Illumination of tissues/cells at a 630–640 nm wavelength results in the formation of reactive oxygen species, including singlet oxygen, which are cytotoxic molecules that damage the cells targeted by PpIX, resulting in cell death (i.e., tumour cells). Intraoperative PDT appears therefore relevant for the treatment of areas adjacent to the resection cavity while maintaining a maximal but safe resection of the GBM.

Over the past decade, there seems to have been a renewed interest for PDT in various medical and surgical areas, translating technical advances in medical lasers and optic fibers that have enabled expanded access to this therapy. However, the few literature reviews carried out in recent years [11–14] report insufficient standardized clinical trials with data and methodology that are difficult to reproduce by other neuro-oncology teams. Some PDT protocols even require technical resources (multi-port lasers) and human resources (engineers, physicists) that are beyond the reach of most teams. This may explain the poor number of multicentre evaluations involving a significant number of patients.

Based on the results of our preclinical data [15–17], non-clinical literature studies [18–20] and previous clinical trials of PDT with 5-ALA [21–24], we initiated a prospective, non-randomized, open-label, pilot study (INDYGO). We now report the long-term results of this standardized and reproducible intraoperative PDT protocol.

### Objectives

The primary objective was to evaluate the feasibility and safety of the intraoperative PDT procedure using a dedicated illumination device. The secondary objectives were to assess the progression free survival (PFS), the overall survival (OS) and health-related quality-of-life (HRQOL).

The design of the study has been described previously [25] and an interim analysis of OS and PFS was published in a previous article [26]. In the present article, we report clinical data obtained during the INDYGO trial and describe the long-term follow-up over 5 years, after the end of the trial period. Moreover, we analysed the impact of treatment on patients' physical, psychological, and social functioning using the EORTC QLQ C30 and BN20 quality of life questionnaires.

## Material and method

### Study design

The INDYGO trial was a prospective, non-randomized, single-centre, open-label, phase I study (NCT 03048240). Patients were eligible if they were 18 years of age, had a newly diagnosed glioblastoma (according to the WHO 2016 classification) with an indication of possible gross total surgical resection of the contrast enhancement. All potential candidate cases were discussed in a multidisciplinary neuro-oncology meeting and inclusion was confirmed. Patients were followed every 3 months until progression or for 30 months, whichever came first.

The study was conducted in accordance with the principles of the Declaration of Helsinki. The protocol was approved by The French National Agency for Medicine and Health Product Safety (ANSM) and reviewed by the French National Ethics Committee. The study was designed in collaboration between the study sponsor and the investigators.

All patients gave written informed consent, according to institutional regulations. The data were analysed and interpreted by a sponsor biostatistician in close collaboration with the investigator. The trial management committee and academic investigator had access to the raw data.

### Setting

Patients were selected and enrolled at a single tertiary neurosurgical centre, the University Hospital of Lille, between May 2017 and June 2018. The last visit of the last included patient took place in April 2021. The database was locked in September 2021 and the study was closed in October 2021. After the end of the clinical trial, patients were followed as part of the standard clinical follow-up and additional survival data were collected.

### Eligibility criteria

Inclusion criteria for the study were as follows: age ≥ 18 years; Karnofsky Performance Status (KPS) score ≥ 60; suggestive cerebral MRI findings suggestive for a HGG; absence of contraindications to MRI; surgical indication; histological diagnosis of glioblastoma; ability to undergo the SOC after surgery; absence of contraindications to MRI; absence of contraindications to 5-ALA HCl (i.e. adequate hepatic and renal function: bilirubin < 1.5 times the upper normal value; serum aspartate aminotransferase, alanine aminotransferases and alkaline phosphatases < 2.5 times normal value; creatinine clearance > 30 mL/min).

Key exclusion criteria included: multifocal disease; hypersensitivity or contraindications of 5-ALA HCl and/or porphyrins; acute or chronic types of porphyria; use of other photosensitizing drugs, history of cardiopulmonary disease; soy allergy; pregnancy or nursing.

### Study participants

12 patients were enrolled, 10 patients with surgically accessible lesions for maximal resection were analysed and 2 were discontinued (one due to a liver dysfunction contraindicating the use of 5-ALA HCl and one due to the intraoperative diagnosis of a left temporal abscess).

One patient at a time was enrolled in the study to allow prospective analysis of safety during and immediately after the procedure. Following the inclusion of the first 5 patients, an independent safety monitoring board (ISMB) met and approved the completion of the trial with the inclusion of five additional patients.

#### Baseline patient characteristics

Baseline patient characteristics are described in Table 1. The median age was 57.1 years [35–69.3], 70% of the patients were male, and the median KPS score was 85 (range 70–100). All patients had a histologically confirmed glioblastoma with IDH mutation present in only one patient (#10). Four patients presented with a hypermethylation of the MGMT promoter gene (#04, #06, #09 and #10).

**Table 1:** Baseline characteristics

Patient	Age (years)	KPS	Gender	Weight (kg)	Tumor Location (lobe/side)	Tumor volume (cm^3^)	% MGMT Methylation	IDH mutation
#1	55.4	70	F	54	Temporal / Left	21	4.50%	No
#2	56.6	100	M	102	Frontal / Right	46.6	2%	No
#3	44.8	90	M	72	Frontal / Right	83.2	1%	No
#4	68.4	80	M	92	Temporal / Right	77.9	18%	No
#5	54.5	90	F	125	Temporal / Right	41.8	4%	No
#6	64.0	70	M	62	Temporal / Right	37.4	31%	No
#7	69.3	80	M	70	Temporal / Left	27.2	4%	No
#8	58.1	90	M	113	Frontal + corpus callosum / Right	78	2%	No
#9	57.6	100	F	68	Temporal / Left	10.5	13%	No
#10	35.0	80	M	83	Frontal / Right	74.6	57.20%	IDH1/IDH2
Median	57.1	85		77.5		44.2		

*F* female, *M* male, *kg* kilograms, *cm* centimeter, *MGMT* O-6-methylguanine-DNA methyltransferase, *IDH* isocitrate dehydrogenases

#### Intraoperative PDT description

**Fig. 1:**
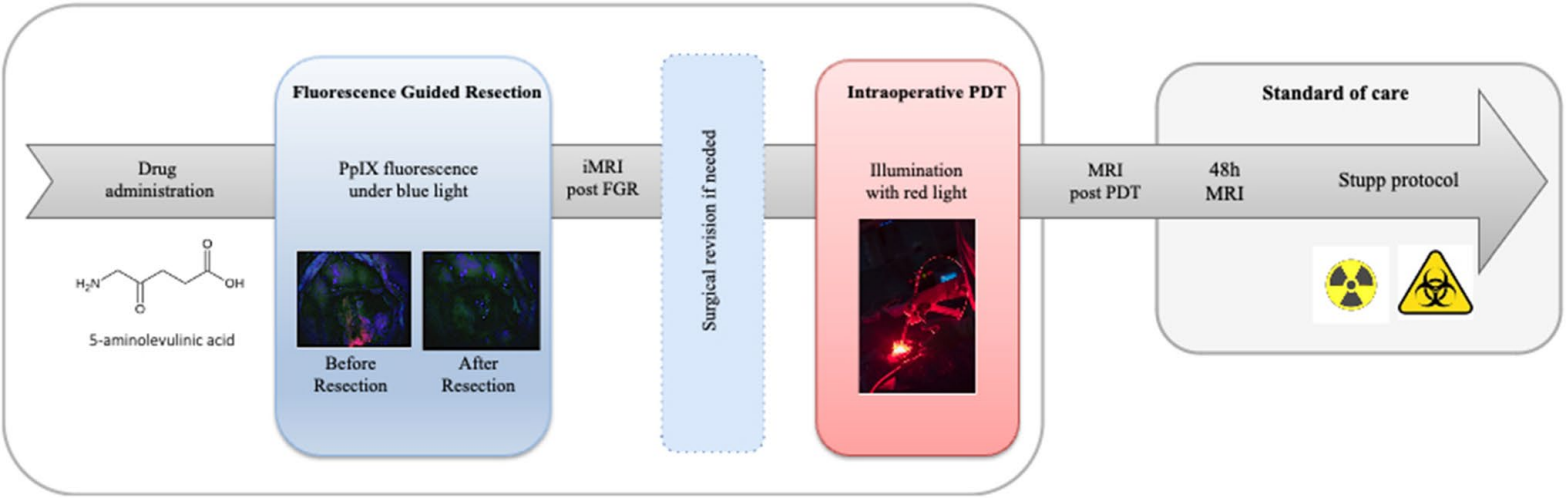
Workflow of the intraoperative PDT procedure; iMRI: intraoperative magnetic resonance imaging, FGR: fluorescence guided resection, PpIX: protoporphyrin IX

Intraoperative PDT procedure (Fig. 1) has been described in a previous article [25]. It consisted of the orally delivery of Gliolan® as photosensitizing drug, a fluorescence-guided gross total resection assessed by intraoperative MRI. Additional resection could then be performed, followed by the illumination of the tumour bed with a specific prototype illumination device. An immediate postoperative MRI was performed early after intraoperative PDT to confirm the absence of an acute adverse event.

**Fig. 2:**
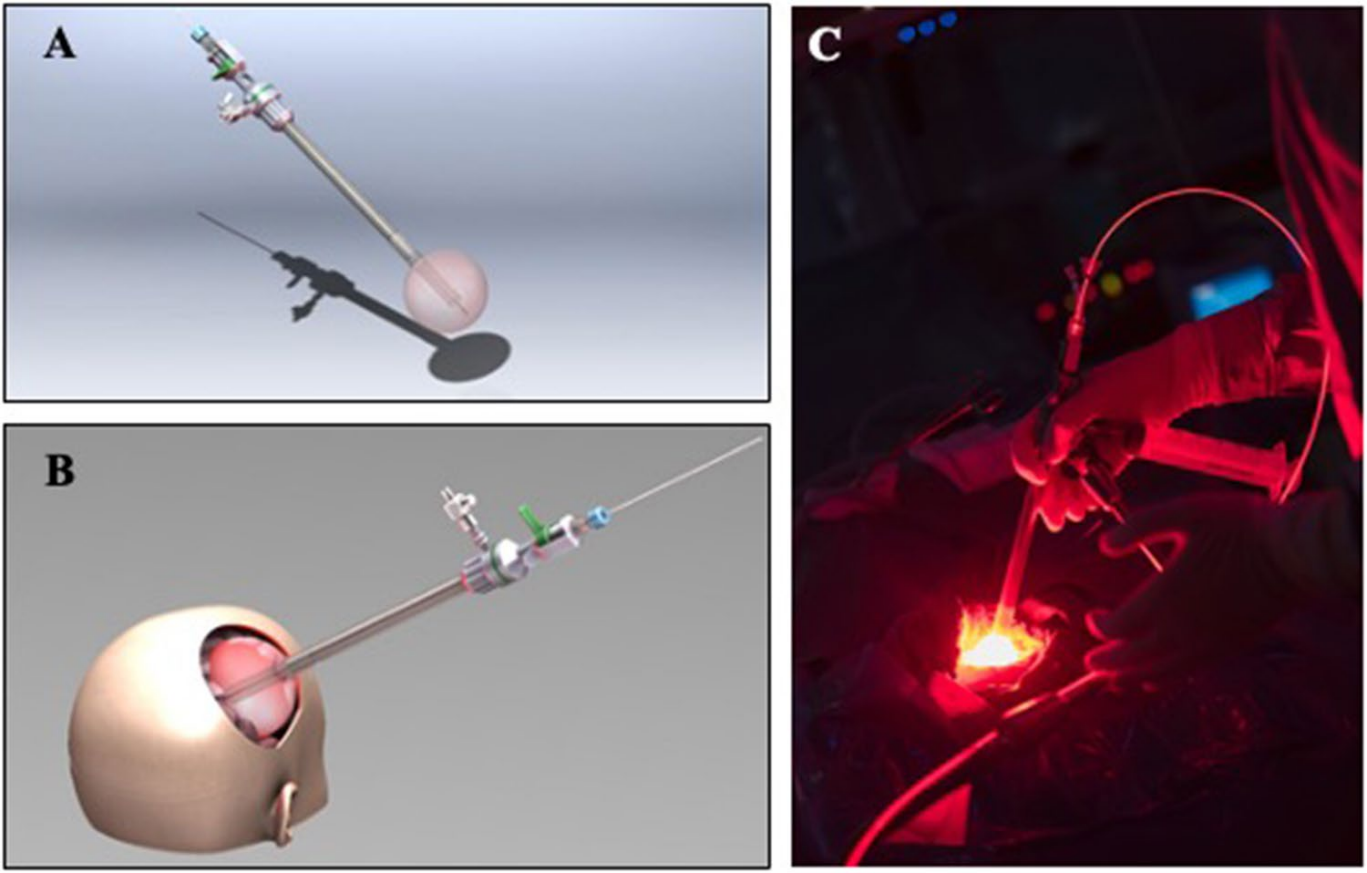
View of the light diffuser (**A**), theoretical (**B**) and intraoperative methodology (**C**) for insertion in the surgical cavity

The illumination device is composed of a red wave-length light diffuser as illustrated in Fig. 2. The distal balloon is inserted and inflated with a intralipid diffusing solution to fit the shape of the resection cavity [27]. Light is emitted through an optical fiber connected to a medical non thermal laser device and diffused within the balloon.

In all patients the total light dose of 200 J/cm^2^ on the balloon surface was delivered in 5 fractions alternating laser on/laser off with an off period of 2 min as evaluated in preclinical experiments [16, 28–30]. This model allows delivery of photodynamic therapy fluence of 25 J/cm^2^ at 5 mm inside surrounding brain tissues as described in a previous paper [27].

All 10 patients underwent an intraoperative MRI (iMRI) using a high-field General Electric Medical System Optima 450MRw machine to assess the initial extent of resection (EOR) before the PDT procedure. The iMRI examination included 3D gadolinium-enhanced T1 (T1Gd) and Fast Imaging Employing Steady-state Acquisition (FIESTA) diffusion and perfusion sequences. In cases where residual contrast enhancement was amenable to further resection, a second stage of microsurgical resection was performed based on the iMRI findings. Once this step was completed, the team proceeded to iPDT illumination per trial protocol. This is expected to induce cell death in any remaining tumour cells.

Surgical characteristics are detailed in Table 2. Five patients underwent total resection of contrast enhancement confirmed by the first intraoperative MRI. Only one of them presented with unresectable vague residual fluorescence in the peri cavitary cerebral parenchyma. Four patients underwent a second stage of microsurgical resection after the first intraoperative MRI. Two patients presented with residual vague fluorescence in eloquent areas with further resection deemed unsafe. Only one patient presented with intense residual fluorescence (no supplementary resection due to eloquent localization).

**Table 2:** Surgical characteristics and iPDT duration

Patient	GTR according to MRI	Second stage of microsurgical resection after iMRI	Illumination time (min) (device setup excluded)
#1	Yes	No	17.78
#2	Yes	No	18.37
#3	Yes	No	24.37
#4	No	Yes	22.60
#5	No	Yes	14.95
#6	Yes	No	17.30
#7	Yes	No	16.48
#8	No	Yes	20.83
#9	Yes	No	14.95
#10	No	Yes	20.83
Median			**18.08**

*iPDT* intraoperative photodynamic therapy, *GTR* gross total resection

At the end of the resection, the surgical cavity was illuminated with a laser using the dedicated illumination device. The duration of the intraoperative PDT procedure (illumination of the surgical cavity) varied according to the volume of the surgical cavity. It ranged from 15 to 25 min with a median total duration of 18 min. Median device setup time was 14 min, for a median total procedure time of 36 min.

#### Adjuvant therapy: the Stupp protocol

After surgery and intraoperative PDT, patients integrated our usual neuro-oncological management: diagnosis confirmation and supplementary information on the standard of care (SOC) for adjuvant treatment. They all accepted and underwent the SOC i.e. concomitant radio-chemotherapy and EANO guidelines [31].

As described in Table 3, all but 1 patient (#09) completed the initial regimen of radiotherapy and concomitant TMZ.

**Table 3:** Stupp protocol adherence

Patient	Total dose RT	Concomitant TMZ (weeks)	Concomitant TMZ (days)	Maintenance TMZ cycles (no.)	Tumor progression during Stupp protocol
#1	60 Gy	6	45	5	Yes
#2	60 Gy	6	43	2	Yes
#3	60 Gy	6	42	5	Yes
#4	60 Gy	6	45	6	No
#5	60 Gy	6	50	6	No
#6	60 Gy	6	43	6	No
#7	60 Gy	6	43	6	No
#8	60 Gy	6	42	4	Yes
#9	60 Gy	4	28	0	No
#10	60 Gy	6	41	6	No

*RT* radiotherapy, *TMZ* temozolomide

Five patients completed the full adjuvant TMZ treatment, 4 patients discontinued maintenance TMZ due to disease progression. Patient #09 did not complete maintenance TMZ due to cytopenia.

### Patients assessment

At least 15 visits were scheduled during patient follow-up. Patients were assessed within 6 weeks of intraoperative PDT then monthly, for 9 months, for medical follow-up and adjuvant therapy, then quarterly until tumour recurrence. Each visit included a full clinical examination and a contrast enhanced MRI with gadolinium-enhanced T1 (T1Gd) and T2/FLAIR sequences to assess treatment response according to the RANO criteria [32].

To evaluate HRQOL and brain tumour-related symptoms, the European Organization for Research and Treatment of Cancer QLQ-30 [33] and QLQ-BN20 [34] questionnaires were used. Participants were asked to complete both questionnaires at the start of the study and every three months for 30 months or earlier if they had a recurrence. HRQOL scores were calculated according to the procedures recommended by EORTC.

The QLQ-C30 questionnaire contains 30 items covering global health, physical status, activity limitation, cognitive, emotional, and social functioning, and occurrence of common symptoms related to cancer or treatment. Raw scores are linearly transformed to 0–100 scales. A high score indicates a better global health, functional status, and greater overall quality of life. A 10% change indicates a significant clinical evolution, and a 20% change will be considered major.

The QLQ-BN20 questionnaire is a 20-item self-reporting tool designed as a supplement to QLQ-C30, to assess HRQOL specifically in brain tumour patients. It includes 20 items assessing disease symptoms (headache, seizures, fatigue, hair loss, skin itching, leg weakness, and bladder control problems) and multi-item scales assessing future uncertainty, visual disturbances, motor dysfunctions, and communication deficits. QLQ-BN20 scores are linearly transformed to a 0–100 scale. A high score indicates greater severity of brain tumour-related symptoms.

For the QLQ-C30 and QLQ-BN20 questionnaires, we calculated the change in the number of points in each score between the first and last questionnaires completed by each patient and then averaged the change in each score across all patients for the duration of the study.

For the safety assessment, all adverse events (AEs) and serious adverse events (SAEs) were collected, evaluated, and graded at each visit. Adverse device effects (ADEs) and serious adverse device effects (SADEs) were also collected during surgery and patient hospitalization. AE and ADE intensity was graded according to NCI-CTCAE V5.0 (Common Terminology Criteria for AEs v5.0).

Special attention was paid to AEs related to 5-ALA HCl (such as emesis, nausea, hepatobiliary disorders, haematological disorders, photo sensibilization) and postoperative complications (such as neurological deficit, severe infection, epileptic status, haemorrhage). AEs and SAEs related to the device or to the PDT procedure were recorded.

### Statistical analysis

For this pilot feasibility study, the number of subjects was not based on statistical assumptions. A total of 10 evaluable patients were enrolled in the study, with the aim that at least 60% of the enrolled patients (i.e., 6 out of 10) would be able to undergo the complete intraoperative PDT without unacceptable and/or unexpected toxicities.

The study is now completed, and the database was locked in September 2021. Five years were calculated from the last included patient and November 2023 was the cut-off date for collecting survival data for the patients still alive.

We have compiled the safety results described in the previous publication [26] and analysed the efficacy and quality of life results until relapse or November 1st, 2023, whichever came first.

Survival analysis from diagnosis to tumour progression, death or last follow-up was plotted using the Kaplan–Meier method (performed using a graphic user interface to the R statistical analysis software for scientific publications, Medistica©, pvalue.io, 2021). Patients were censored at the time of last follow-up or at the time of death.

## Results

### Safety endpoints

During the trial, the majority of AEs were due to chemotherapy and radiotherapy (59/167), to unspecified causes, (14/167), to the surgery without relation to the PDT procedure, and/or were GBM-related symptoms (13/167). They were graded 1 or 2; 10 AEs were graded 3 according to CTCAE v5.0.

4 AEs were related to the study procedure, specifically to 5-ALA HCl intake (1 erythema [grade 2], 3 liver enzyme increase [two grade 2, one grade 1]). These events were expected with the use of 5-ALA HCl.

7 SAEs were reported in 5 patients, including one grade 5 SAE leading to patient death. But all SAEs were unrelated to the PDT procedure.

After the Stupp protocol, patient #09 experienced partial status epilepticus revealing a remote relapsing glial lesion leading to death due to tumour progression.

Finally, no adverse device effect was reported after the use of the device.

Regarding the safety data, the ISMB concluded that the addition of the PDT procedure during surgery in patients with newly diagnosed glioblastoma was not associated with any significant adverse or toxic effects.

## Efficacy endpoints

**Fig. 3:**
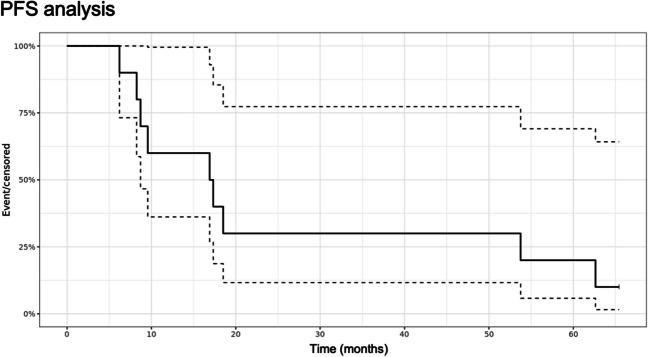
Kaplan–Meier curve for PFS (median values represented by solid black line, 25–75 confidence interval represented by dotted lines)

**Fig. 4:**
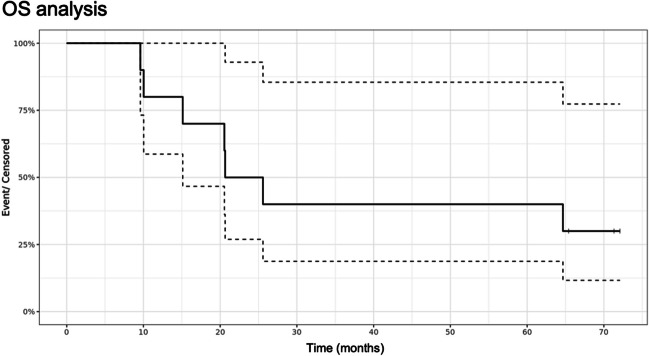
Kaplan–Meier curve for OS (median values represented by solid black line, 25–75 confidence interval represented by dotted lines)

Tables 4 and 5 describe overall PFS and OS results. Figures 3 and 4 demonstrate the Kaplan–Meier curves for PFS and OS, respectively.

**Table 4:** PFS and OS overview

Patient	Age (years)	Preoperative KPS	PFS (months)	OS (months)
#1	55.4	70	9.57	20.64
#2	56.6	100	6.21	10.02
#3	44.8	90	8.25	15.12
#4	68.4	80	17.33	20.54
#5	54.5	90	18.48	25.57
#6	64.0	70	62.63	*72.09*^*2*^
#7	69.3	80	53.75	*71.30*^*2*^
#8	58.1	90	8.71	9.6
#9	57.6	100	16.90	64.66
#10	35.0	80	^1^	*65.42*^*2*^
Median	**57.1**	**85**	**17.11**	**23.11**

*KPS* Karnofsky performance score, *PFS* progression-free survival, *OS* overall survival, ^1^ no recurrence at the latest follow- up in November 2023; ^2^ still alive at the latest follow- up in November 2023

**Table 5:** Summary of INDYGO efficacy results

10 patients treated	6 months	12 months	24 months	5 years
PFS	100%	60%	30%	20%
OS	100%	80%	50%	40%

At 6 months, PFS was 100%, no patient showed recurrence. Early recurrences before 9 months were discovered during the Stupp protocol in 3 patients (#02, #03 and #08).

At 12 months, 6 out of 10 patients had no recurrence and at the date of this analysis, 4 patients were still alive including 1 patient without recurrence.

## Evolution of the quality of life

As described in Table 6, the median “Global Health Status” score of patients improved between the first and last visits. The median scores of all patients on the dysfunction scales (visual, motor, and cognitive) and symptoms experienced (fatigue, nausea, pain, insomnia, etc.) remained stable or improved.

**Table 6:** Variation of the QLQ-C30 and QLQ-BN20 scores

Items	Mean	Median
EORTC QLQ-C30		
Global Health Status	13.89	+ *25.00*
Physical functioning scale	10.00	0.00
Activity& leisure functioning scale	26.67	+ *25.00*
Emotional functioning scale	16.39	+ *12.50*
Cognitive functioning scale	8.33	0.00
Social functioning scale	10.00	0.00
Fatigue	-16.67	*-11.11*
Nausea & vomiting	-5.00	0.00
Pain	-16.67	*-16.67*
Dyspnea	-6.67	0.00
Insomnia	-10.00	*-16.67*
Appetite loss	-13.33	0.00
Constipation	-3.33	0.00
Diarrhea	-6.67	0.00
Financial difficulties	0.00	0.00
EORTC QLQ-BN20		
Future Uncertainty	17.50	+ *16.67*
Visual Disorders	10.56	+ *5.56*
Motor dysfunction	1.11	0.00
Cognitive dysfunction	11.11	0.00
Headache	-13.33	0.00
Seizures	-11.11	0.00
Drowsiness	-6.67	0.00
Itchy skin	8.33	0.00
Hair Loss	-9.52	0.00
Weakness of legs	7.41	0.00
Bladder control	16.67	0.00

The overall analysis of the questionnaires seemed to show a stability in disease symptoms and a preservation of the functional scores, but these were not significant given the small number of patients who remained in the study beyond 9 months of follow-up.

## Case illustration patient #07

**Fig. 5:**
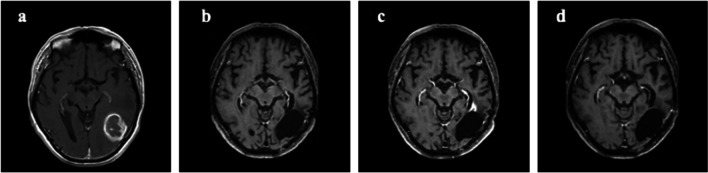
Case illustration of a 70-year-old patient (patient #07) presenting with a left temporo-occipital tumor; 5a: preoperative axial T1 gadolinium-enhanced cerebral MRI; 5b: postoperative MRI at 51 months follow-up showing no recurrence; 5c: postoperative MRI at 55.5 months follow-up confirming tumor recurrence; 5d: postoperative MRI at 67.5 months follow-up showing control of the disease after 12-cycle chemotherapy with temozolomide

Patient #07 underwent surgery and intraoperative PDT in February 2018 (Fig. 5). Histomolecular analysis after resection and intraoperative PDT confirmed a IDH wild-type glioblastoma with 4% methylation of MGMT gene promoter, gain of chromosome 7, loss of chromosome 10, CDKN2A mutation, EGFR amplification. Figure 5b shows no recurrence at 51 months post iPDT but recurrence was confirmed 4 months later at 55 months from the iPDT (Fig. 5c) in October 2022. At the time of the report, patient #07 was still alive at 69 months after diagnosis, despite negative prognostic factors: elderly patient, IDH wild-type, absence of MGMT gene promoter methylation, CDKN2A mutation, and EGFR amplification.

## Adjuvant treatment after recurrence

Table 7 demonstrates location of initial tumour and differences in locations for glioblastoma relapse. Second and third lines of treatment are also detailed. Noteworthy, 7 out of 10 patients relapsed before 30 months. Four patients were still alive at the end of the study.

**Table 7:** Initial tumor location, recurrence location, second and third lines of treatment

Patient	Location	Relapse	PFS in months	Second / third lines treatment post relapse
#01	Left temporal	Remote	9.57	radiation therapy (3 × 9 Gy) + lomustine / bevacizumab
#02	Right frontal	Remote	6.21	radiation therapy (60 Gy) + temozolomide / bevacizumab + lomustine
#03	Right frontal	Local	8.25	bevacizumab + fotemustine
#04	Right temporal	Local	17.33	temozolomide
#05	Right temporal	Remote then local progression	18.48	bevacizumab + lomustine depatuxizumab mafodotin + temozolomide
#06	Right temporal	Local	62.63	radiation therapy (10 × 3.5 Gy) + bevacizumab + temozolomide
#07	Left temporal	Local	53.75	temozolomide
#08	Right frontal	Local	8.71	bevacizumab + lomustine
#09	Left Temporal	Local	16.9	resurgery + carmustine implant + temozolomide
#10	Right frontal		No	

## Discussion

Treatment within the field of malignant gliomas is evolving to emphasize combined approaches that work synergistically to improve patient outcomes. Thus, having multiple mechanisms of action (necrosis, apoptosis, immunostimulation) makes intraoperative PDT an attractive modality to fluidly include in the standard and novel treatment regimens (e.g., maximal resection, radiotherapy, chemotherapy, immunotherapy or TTFields).

The INDYGO study was designed to evaluate the feasibility and the safety of adding intraoperative PDT to standard treatment e.g., surgery + Stupp protocol, in newly diagnosed patients. We reported the results of the study in a previous paper [26] with ultimately no unacceptable or unexpected toxicities after 5-ALA PDT. Another objective was to evaluate the academic prototype of an illumination device developed by our laboratory for intraoperative PDT application [27]. This device enabled dosimetry by automatic calculation of the illumination time according to the volume of the balloon adapted to the operating cavity. This tool was further developed with the aim of being easily used by any neurosurgical team, without the need for human resources other than the habitual surgical team. In particular, it does not require medical physicists or dosimetrists. Thanks to the short installation time and ease of use, this device appears ideally suited to intraoperative PDT.

In this paper we focus on reporting the quality of life and the long-term results. After a median follow-up of 23 months, we observed a 17-month median PFS, from diagnosis to radiological recurrence on T1-weighted post-gadolinium MRI, and a 23-month median OS from diagnosis. Decraene B. et al. [35] define long-term survival in GBM patients as those who survive at least 5 years (60 months) after diagnosis. With respectively a 2-year and 5-year survival rate of 50% and 40%, our therapy seems to hold the promise of significantly prolonging survival.

Interestingly, only 66% of tumour recurrence sites (6/9) were located adjacent to the tumour bed, whereas in published reports 85% of tumour recurrence sites are commonly identified at or near the resection margins [8]. Among our patients, patient #10 may be questionable. This 35-year-old patient, who did not relapse, presented with a grade 4 IDH-mutant astrocytoma. This tumour is no longer graded as a glioblastoma since the 2021 WHO classification of tumours of the central nervous system. It was considered as an IDH-mutant glioblastoma at the time of the INDYGO study (2016 WHO classification). Moreover, the young age of the patient can be regarded as another favourable prognosis factor, being the youngest included patient in this study.

Analysing the results of our PDT protocol as an add-on technique to the standard of care, the results of this study should be compared to patient series managed by standard of care alone. This comparison obviously has to be considered cautiously due to the size of our population.

The study by Gerritsen et al. [36] reports, after maximal extent of resection in 4 groups of GBM patients (aged < 70 years old), a 9.3-month PFS and a 19.5-month OS. The recent RESECT study [37] actually provides an interesting comparison with our study. This randomized multicenter study compared 5-ALA fluorescence-guided surgery (FGS) with white-light surgery in patients presenting with newly diagnosed glioblastoma. This study provides a population of patients who underwent FGS alone, and the INDYGO population underwent FGS associated to PDT. In the 5-ALA FGS group, the RESECT study reported a 10.0-month median PFS 95% CI (8.5–11.9 months)), an 18.7 -month median OS (95% CI (17.1–22 months)) and a 24-month OS rate of 30.1% [95% CI 18.9%–42.0%]. The study by Stupp et al. [38], which assessed the impact of tumor-treating fields (TTFields), another add-on technique, reported 6.7 months median PFS from randomization in the TTFields-temozolomide group and a 20.9 months median overall survival in the TTFields-temozolomide group. The 2 and 5-year survival actuarial rates were 40% and 13% respectively. Regarding these studies, the long-term results of INDYGO appear promising particularly regarding the PFS and the long-term survival rates.

Another intraoperative therapy that could be compared to our study is the INTRAGO trial analysing image-guided radiotherapy with dose-escalation after glioblastoma resection [39]. In a cohort of 15 patients, Giordano et al. showed a median PFS of 11.3 months (local-PFS of 17.8 months for patients receiving per-protocol treatment). Among side effects authors described radiation necrosis (5 patients), wound dehiscence (1 patient), CSF leakage (1 patient), and cyst formation (1 patient). In contrast, the use of the iPDT device showed no immediate significant adverse or toxic effects. At 5-years follow-up, no post-therapeutic necrosis was observed after iPDT at 200 J/cm^2^, neither before nor after the completion of concomitant radio-chemotherapy standard protocol. This should be evaluated on larger cohorts to confirm these encouraging results on long-term toxicity.

Regarding quality of life, the application of the PDT intraoperatively, in a single session, shortly after tumour removal, had no impact on patient management. No further treatment or specific follow-up was required and quality of life appeared to be maintained or improved in a small number of patients. This quality of life is probably related to the increased PFS delaying second line treatment.

Considering the results of this study in terms of survival and safety, it seemed appropriate to our team to evaluate this intraoperative PDT protocol with an escalation of the light dose. To date, enrolment in the DOSINDYGO study (NCT04391062), a second trial of intraoperative PDT with a dose escalation, is closed. Light doses of 400 J/cm^2^, 600 J/cm^2^ and 800 J/cm^2^ were successfully applied in 13 patients. The safety results are currently evaluated*.*

The standardized nature of this intraoperative PDT protocol now allows for other teams to evaluate it.

Noteworthy, the drug Pentalafen® (5-ALA HCl) and a new version of the device, Heliance® Solution (Hemerion Therapeutics, France), will be evaluated in a first-in-human phase 1 study of intraoperative PDT in glioblastoma patients starting in the US early 2024 (IND #16491- NCT: NCT05736406).

## Limitations

The results of the study are limited by the absence of patient randomization and the small number of patients enrolled in a single center. Moreover, the study was designed to evaluate the safety of the intraoperative procedure, not its efficacy. Nevertheless, the fact that we followed the patients over a long period of time enabled us to monitor their quality of life and the standard parameters for tumour evolution (PFS and OS).

## Conclusion

The INDYGO study reported on a standardized and reproducible intraoperative PDT protocol. This study also confirms the possibility of introducing safe photodynamic therapy treatment easily into an operating ward during surgery.

Taking into account the small reported cohort this clinical trial results suggest improved progression-free survival (PFS) and overall survival (OS) rates compared to patients treated with the standard of care. These positive outcomes require confirmation through an evaluation on a larger cohort of glioblastoma patients or during a randomised trial. The intraoperative PDT protocol is now mature enough to be applied across multiple centers.

## Data Availability

No datasets were generated or analysed during the current study.

## Code availability

Not applicable for that section.
